# LPS‐induced inflammation desensitizes hepatocytes to Fas‐induced apoptosis through Stat3 activation—The effect can be reversed by ruxolitinib

**DOI:** 10.1111/jcmm.14930

**Published:** 2020-02-05

**Authors:** Antonio Markotic, Darja Flegar, Danka Grcevic, Alan Sucur, Hrvoje Lalic, Petra Turcic, Natasa Kovacic, Nina Lukac, Danijel Pravdic, Katarina Vukojevic, Ivan Cavar, Tomislav Kelava

**Affiliations:** ^1^ Laboratory for Molecular Immunology Croatian Institute for Brain Research School of Medicine University of Zagreb Zagreb Croatia; ^2^ Center for Clinical Pharmacology University Clinical Hospital Mostar Mostar Bosnia and Herzegovina; ^3^ Department of Physiology School of Medicine University of Zagreb Zagreb Croatia; ^4^ Department of Pharmacology Faculty of Pharmacy and Biochemistry University of Zagreb Zagreb Croatia; ^5^ Department of Anatomy School of Medicine University of Zagreb Zagreb Croatia; ^6^ Department of Physiology School of Medicine University of Mostar Mostar Bosnia and Herzegovina; ^7^ University Clinical Hospital Mostar Mostar Bosnia and Herzegovina; ^8^ Department of Anatomy, Histology and Embryology School of Medicine University of Split Split Croatia; ^9^ Department of Medical Genetics School of Medicine University of Mostar Mostar Bosnia and Herzegovina

**Keywords:** apoptosis, Fas, lipopolysaccharide, ruxolitinib, Stat3

## Abstract

Recent studies have established a concept of tumour necrosis factor‐α (TNF‐α)/Fas signalling crosstalk, highlighting TNF‐α as a critical cytokine in sensitizing hepatocytes to death induced by Fas activation. However, in the exact inflammatory response, besides TNF‐α, many other mediators, that might modulate apoptotic response differentially, are released. To resolve the issue, we studied the effects of lipopolysaccharide (LPS), one of the crucial inductors of inflammation in the liver, on apoptotic outcome. We show that LPS‐induced inflammation diminishes the sensitivity of hepatocytes to Fas stimulus in vivo at caspase‐8 level. Analysis of molecular mechanisms revealed an increased expression of various pro‐inflammatory cytokines in non‐parenchymal liver cells and hepatocyte‐specific increase in Bcl‐xL, associated with signal transducer and activator of transcription 3 (Stat3) phosphorylation. Pre‐treatment with ruxolitinib, a selective Janus kinase (JAK) 1/2 inhibitor, prevented the LPS‐induced Stat3 phosphorylation and restored the sensitivity of hepatocytes to Fas‐mediated apoptosis. Furthermore, ruxolitinib pre‐treatment diminished the LPS‐induced Bcl‐xL up‐regulation without an inhibitory effect on LPS‐induced expression of pro‐inflammatory cytokines. In summary, although the reports are showing that the effects of isolated pro‐inflammatory mediators, such as TNF‐α or neutrophils, are pro‐apoptotic, the overall effect of inflammatory milieu on hepatocytes in vivo is Stat3‐dependent desensitization to Fas‐mediated apoptosis.

## 1 INTRODUCTION

Several recent studies have established a concept of tumour necrosis factor‐α (TNF‐α)/Fas signalling crosstalk, highlighting TNF‐α as a critical cytokine in sensitizing hepatocytes to death induced by Fas (CD95) activation.^1^, ^2^, ^3^, ^4^ However, in the exact inflammatory response, besides TNF‐α, many other soluble and cellular mediators, such as interleukin (IL)‐6, IL‐1β, neutrophils, natural killer (NK) and NKT cells, are induced, that might modulate apoptotic response differently.^5^, ^6^, ^7^, ^8^ Numerous studies on the effects of isolated mediators on Fas pathway in hepatocytes provided valuable insight into the complex crosstalk between the pro‐survival and pro‐apoptotic signals in liver cells.^1^, ^2^, ^9^, ^10^, ^11^, ^12^ However, focusing on one isolated mediator, even on paramount pro‐inflammatory one, like TNF‐α, might blur the understanding of the overall effect of inflammatory milieu on apoptosis.

The disbalance in apoptotic or inflammatory processes is thought to be a critical event in the pathogenesis of various hepatic diseases such as viral hepatitis, cirrhosis and hepatocellular carcinoma (HCC).^6^, ^13^, ^14^, ^15^, ^16^, ^17^ Therefore, the correct assessment of their complex interplay is crucial for the development of novel and effective treatment‐ or prevention‐oriented strategies. Recent work from Burdelya et al^8^ has shown that Toll‐like receptor 5 (TLR5) agonist CBLB502 protects mice from Fas‐mediated hepatotoxicity. However, the principal cause of liver inflammation is gut‐derived lipopolysaccharide (LPS), a prototypical TLR4 agonist.^5^ Growing evidence suggests that gut‐derived LPS affects the extent of liver damage or even plays a crucial role in certain diseases such as alcoholic liver disease and HCC.^18^, ^19^ Contrary to TLR5 agonists, LPS induces high expression of TNF‐α, whose pro‐apoptotic effects through crosstalk with the Fas pathway have been in the focus of recent research.^1^, ^2^, ^3^, ^8^

In the present study, we analyse LPS‐induced effects on downstream events of cytokine signalling in the liver and show that LPS‐induced inflammation has a powerful anti‐apoptotic effect on Fas‐induced liver injury. We identify phosphorylation of signal transducer and activator of transcription 3 (Stat3) as a key downstream mechanism in desensitizing hepatocytes to apoptotic death and show that anti‐apoptotic effect of inflammatory milieu can be abrogated by ruxolitinib‐induced Janus kinase (JAK) 1/2 inhibition.

## 2 MATERIALS AND METHODS

### 2.1 Mice

Male C57BL/6 mice, 20‐25 grams bodyweight, were kept under standard conditions in the animal facility of the Croatian Institute for Brain Research. All experiments and experimental protocols were approved by Directorate for Veterinary and Food Safety of the Ministry of Agriculture. Experiments were designed and conducted in concordance with the 3R's principles.

### 2.2 Dosage and application routes

We injected intraperitoneally 0.1 mg/kg of LPS (Sigma‐Aldrich) dissolved in sterile saline to induce acute inflammation unless otherwise stated.

To induce apoptosis, we dissolved the anti‐Fas agonistic antibody (hamster antimouse CD95, clone Jo2; BD Pharmingen, San Diego, CA, USA) in sterile phosphate buffered saline (PBS) and injected it intravenously (0.25 mg/kg).

A specific inhibitor of liver transcription D‐galactosamine (D‐GalN; Sigma‐Aldrich) was dissolved in saline and injected intraperitoneally (700 mg/kg).

Ruxolitinib (INCB018424; Selleckchem, Houston, TX, USA), a selective JAK1/2 inhibitor, was used to suppress Stat3 phosphorylation. Ruxolitinib was dissolved according to the manufacturer's instructions with slight modifications (3% dimethyl sulfoxide [DMSO] + 30% polyethylene glycol 300 [PEG300] + distilled water [dH2O]), sonicated for 1 hour and applied by oral gavage two times (with 2 hours between applications) to a final dose of 180 mg/kg.

Avertin (2.5%) was applied intraperitoneally (0.25 mg/g) for anaesthesia.

### 2.3 Experimental design

In the first set of experiments, animals were treated with LPS or saline, followed by anti‐Fas 2 hours later, to assess the effect of acute inflammation on apoptosis. The control group received vehicles (n = 4‐6 per group). After an additional 6 hours, blood and liver samples were collected for transaminase activity measurements, caspase‐8 activity assay and pathohistological analysis.

In the second set of experiments, we analysed changes that occur in the liver following LPS treatment. To investigate changes in non‐parenchymal cell populations, we treated mice with LPS or saline, and after 2 hours, whole livers were processed for flow cytometry analysis. The same protocol was used to obtain serum for ELISA and liver tissue samples for immunohistochemistry. For gene expression analysis, mice were treated with LPS alone, D‐GalN/LPS or saline. Blood and liver tissue specimens were collected 90 minutes after the treatment (n = 5 per group). D‐GalN is a specific inhibitor of transcription in hepatocytes, and it was used to distinguish whether the observed changes in gene expression were in hepatocytes or non‐parenchymal liver cells.^20^

In the final experiments, the effect of ruxolitinib on the model was examined. Mice received ruxolitinib or vehicle (DMSO + PEG300 + dH2O) 4 and 2 hours before LPS (0 hour), which was followed by anti‐Fas antibody after 2 hours. Control groups were as follows: ruxolitinib/saline/anti‐Fas, DMSO + PEG300 + dH2O/saline/anti‐Fas, ruxolitinib/saline/PBS, ruxolitinib/LPS/PBS and DMSO + PEG300 + dH2O/LPS/PBS (n = 3‐8 per group). Six hours after anti‐Fas application, serum and liver samples were collected. For Western blot analysis of phosphorylated Stat3 (pStat3), immunohistochemistry and PCR, ruxolitinib or vehicle with LPS were applied as mentioned before, and liver samples were collected 90 minutes after the LPS treatment.

### 2.4 Transaminase activity assay

Alanine aminotransferase (ALT) and aspartate aminotransferase (AST) activities in serum were measured by standard laboratory method as described previously.^21^

### 2.5 Caspase‐8 activity assay

Caspase‐8 activity was determined by The ApoTarget™ Caspase‐8 Protease Assay reagent set (Invitrogen), according to manufacturer's instructions.

### 2.6 Gene expression analysis

RNA was isolated from the liver after homogenization in TRIzol (Invitrogen), reversely transcribed into complementary DNA using High Capacity RNA‐to‐cDNA Kit (Applied Biosystems) and amplified by the real‐time qPCR procedure using ABI PRISM 7500 Sequence Detection System (Applied Biosystems). TaqMan assays (IDs in Supporting Information) were used to determine the expression of TNF‐α, IL‐6, IL‐1β, Fas, Bcl‐2, Bcl‐xL, CFLAR, XIAP, cyclin D1 and survivin with GAPDH as the housekeeping gene. SYBR green real‐time qPCR in conjunction with primers for soluble Fas (FasB) and β‐actin was utilized.^22^ All comparisons between groups were done at the ΔCt level and plotted as a fold difference (2^−ΔΔCT^ method) with 95% CI against the vehicle‐treated group.^23^

### 2.7 Isolation of liver non‐parenchymal cells and flow cytometry

Mice were anesthetized, livers perfused with sterile PBS, and non‐parenchymal liver cells isolated as previously described (with slight modifications).^24^ Briefly, after successful perfusion, whole livers were excised and minced, incubated with 0.2 mg/mL collagenase IV buffer at 37°C for 30 minutes with shaking, double‐passed through 70 μm nylon mesh and centrifuged twice for 3 minutes at 40 *g* to pellet hepatocytes. Supernatants containing non‐parenchymal cells were purified in the Percoll gradient (Sigma‐Aldrich). Isolated cells were counted in hemocytometer (Bürker‐Türk chamber) and labelled with fluorescent‐conjugated antibodies: CD45‐APC (eBioscience), CD3‐APCeF780 (eBioscience), B220‐PECy7 (eBioscience), NK1.1‐PE (eBioscience), CD11b‐PECy7 (eBioscience), F4/80‐APCeF780 (eBioscience), Ly6G‐PE (BD Pharmingen) and CD95‐AF488 (eBioscience). CD16/CD32 (eBioscience) was used for blocking of Fc receptors, while dead cells were excluded based on 7‐amino‐actinomycin D (7‐AAD; BioLegend) binding. Upon labelling, cells were acquired and analysed using the Attune instrument (Thermo Fisher Scientific) and FlowJo software (FlowJo). The gating strategy is shown in Supporting Information (Figure S1).

### 2.8 ELISA

Soluble Fas concentration in serum was measured by ELISA using commercially available Mouse sFAS ELISA kit (Novatein Biosciences) per manufacturer's instructions.

### 2.9 Histology and Immunohistochemistry

Liver tissue samples were stored in 4% buffered paraformaldehyde, dehydrated in an alcohol gradient and embedded in paraffin, and sections were cut at 5 μm thickness. After deparaffinization in xylol and rehydration, sections were stained with haematoxylin‐eosin, and liver architecture and apoptotic hallmarks were analysed under the microscope (Axiovert 200; Carl Zeiss).

To confirm DNA fragmentation and apoptosis after the anti‐Fas antibody application, we examined the staining of hepatocyte nuclei by the nick translation method, using a previously described protocol with slight modifications.^25^ Briefly, for antigen retrieval, rehydrated sections were cooked in sodium citrate buffer in a microwave oven (20 minutes at 95°C). Upon cooling at room temperature, sections were incubated for 3 hours at room temperature in a labelling mixture containing the following: dATP, dGTP, dCTP and biotin‐16dUTP, DNA polymerase I (Roche) and β‐mercaptoethanol, all mixed in NT buffer containing MgCl_2_, Tris‐Cl and BSA. Slides were then washed with PBS and incubated for 30 minutes in the dark with staining buffer: 4x SSC, Avidin‐FITC, milk powder and Triton X‐100. Sections were washed, counterstained with diamidino‐2‐phenylindole (DAPI) and analysed under a fluorescent microscope (Axiovert 200; Carl Zeiss).

Immunohistochemistry was used to evaluate the processing of caspase‐3 following the anti‐Fas treatment, for the confirmation of the changes in neutrophil number in liver tissue and for the detection of an increase in pStat3 signal in hepatocyte nuclei. The changes in the neutrophil number and pStat3 signal were both determined 2 hours after LPS treatment. Briefly, mice were anesthetized, and livers were perfused using sterile PBS and 4% paraformaldehyde, respectively. Sections were prepared as described before.^21^

For neutrophil detection, slides were incubated overnight with diluted (1:200) polyclonal rabbit antimyeloperoxidase (MPO) antibody (Dako, Denmark, #A0398). Following multiple washes, sections were stained with diluted (1:300) donkey antirabbit secondary antibody (Abcam, #ab150073) for 1 hour at room temperature, washed again and counterstained with DAPI. For cleaved caspase‐3 and pStat3 detection, rabbit monoclonal anticleaved caspase‐3 and anti‐phospho‐Stat3 antibodies (Cell Signaling Technology, #9664 and #9145, respectively) with rabbit horseradish peroxidase (HRP) SignalStain Boost IHC Detection Reagent (Cell Signaling, #8114) were used according to the protocol provided on the manufacturer's website. Slides were analysed under a fluorescent microscope (Axiovert 200; Carl Zeiss).

### 2.10 Isolation of total cell lysates and Western blot analysis

Mice were killed, and approximately 50 mg of liver tissue was excised and immersed in lysis buffer (Cell Signaling, #9803) enriched with Halt Protease (Thermo Fisher, #87786) and Halt Phosphatase Inhibitor Cocktail (Thermo Fisher, #78420), followed by immediate homogenization. Samples were centrifuged (14 000 *g*, 10 minutes, 4°C), supernatants were collected, and the protein concentration was determined using Bradford protein assay. Supernatants were then mixed with NuPAGE^®^ LDS sample buffer and NuPAGE^®^ reducing agent (Thermo Fisher, #NP0007 and #NP0009, respectively) and denaturated (5 minutes, 95°C). Equal amounts of proteins in each sample (50 μg) were loaded onto 12% stain‐free gels prepared with TGX Stain‐Free^TM^ FastCast Acrylamide Kit (Bio‐Rad Laboratories) and separated by PAGE‐SDS electrophoresis. Gels were activated (5 minutes/UV), which was followed by proteins transfer onto the 0.45 µm methanol‐activated PVDF membrane. Following the short UV excitation, the total protein amount was assessed by membrane imaging. After transfer, membranes were blocked for 1 hour in 0.1% PBS‐Tween buffer containing 5% non‐fat dried milk, washed three times in PBS‐Tween and incubated overnight at 4°C with primary antibodies diluted (1:1000 for Stat3, pStat3 or Bid) in PBS‐Tween containing 5% non‐fat dried milk. Separate membranes were used for the Stat3 and pStat3 immunoblotting. After incubation, membranes were washed and incubated for 120 minutes at RT with secondary antibodies diluted in PBS‐Tween. Membranes were then washed, and the antibody binding was detected with SuperSignal™ West Femto Maximum Sensitivity Substrate (Thermo Fisher) using imaging system ChemiDoc XRS+ (Bio‐Rad). Image Lab Software (Bio‐Rad) was utilized for signal intensity quantification and normalization to total protein signal intensity. Antibodies against Stat3 (#4904), phosphorylated (Tyr705) Stat3 (#9145) and antirabbit IgG (#7074) conjugated to HRP were purchased from Cell Signaling, the antibody against Bid from R&D systems (Minneapolis, #MAB860), and the antirat IgG antibody conjugated to HRP was purchased from Jackson ImmunoResearch. The stain‐free technology for total protein staining was used as a loading control.^26^ For convenience, the stain‐free gels, total protein membranes and the protein of interest membranes are provided in the Supporting information.

### 2.11 Statistical analysis

Data are presented as median with individual values or mean ± SEM and analysed using Student's *t* test/ANOVA or Mann‐Whitney test, as appropriate. Post hoc tests and multiple comparisons were Bonferroni corrected, and adjusted *P* values are reported. All tests were two‐sided, and *P* < .05 was considered statistically significant. GraphPad Prism version 6 for Windows (GraphPad Software Inc) software was used for analysis.

## 3 RESULTS

### 3.1 TLR4 agonist LPS alleviates liver damage induced by activating anti‐Fas antibody

**Figure 1:**
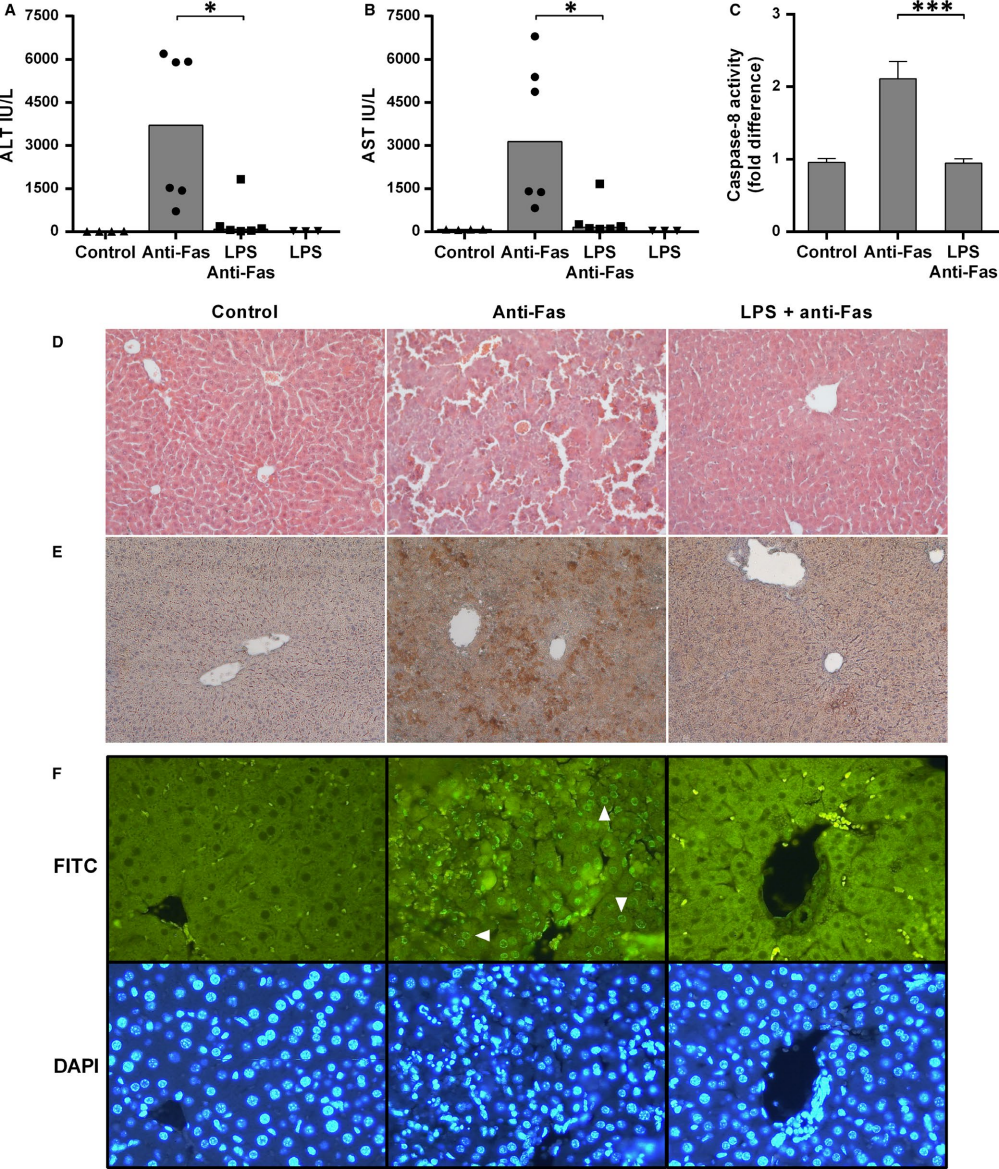
LPS treatment protects hepatocytes from Fas‐induced apoptosis. Mice were treated with LPS (0.1 mg/kg) or saline, and after 2 h, activating anti‐Fas antibody (0.25 mg/kg) or vehicle (PBS) was injected. The control group received vehicles (saline and sterile PBS). Blood samples and liver specimens were collected 6 h after anti‐Fas treatment. A, B, ALT and AST serum levels in LPS pre‐treated group are significantly lower than in saline pre‐treated mice. Symbols represent individual values, and columns represent medians; Mann‐Whitney test. C, Caspase‐8 activity was determined in liver tissue specimens using commercially available colorimetric assay. Columns and bars represent mean ± SEM; Student's *t* test. D, Liver sections were stained with haematoxylin‐eosin for histological assessment. LPS pre‐treated mice preserved normal liver architecture, while there are an obvious injury, haemorrhage and loss of normal liver architecture in the anti‐Fas group. E, Immunohistochemical detection of cleaved caspase‐3 in liver sections. Anti‐Fas antibody induced the expression of cleaved caspase‐3 in livers, as shown by brown staining of liver tissue. Little or almost no processing of the caspase‐3 was observed in LPS pre‐treated animals. F, Nick translation assay was used to confirm DNA fragmentation and apoptosis. White arrowheads show positive staining of hepatocytes nuclei following anti‐Fas treatment in saline pre‐treated animals. In comparison, there was no such signal in LPS pre‐treated group or control mice. DAPI was used as counterstain. n = 4‐6 per group; *P < .05, ***P < .001 (saline/anti‐Fas vs LPS/anti‐Fas). ALT, alanine aminotransferase; AST, aspartate aminotransferase; DAPI, diamidino‐2‐phenylindole; FITC, fluorescein isothiocyanate; LPS, lipopolysaccharide; PBS, phosphate buffered saline

To examine the effect of liver inflammation on the apoptotic response, we treated mice with LPS or saline, which was followed by the injection of activating anti‐Fas antibody after 2 hours. Contrary to previous hypotheses and expectations,^2^, ^3^ LPS desensitized hepatocytes to Fas‐mediated apoptosis and significantly reduced liver damage by the anti‐Fas antibody. LPS pre‐treated group had significantly lower levels of ALT and AST when compared to saline pre‐treated mice (*P* = .015 for both, Figure 1A,B). The histological assessment confirmed these findings, with LPS pre‐treated group exhibiting nearly normal liver architecture, almost without cleaved caspase‐3 expression, while there was an apparent liver injury, caspase‐3 processing and DNA fragmentation in saline/anti‐Fas‐treated group (Figure 1D‐F).

We also tested caspase‐8 activity in the liver in order to ascertain at which level the Fas pathway is blocked by inflammation. Caspase‐8 activity was increased after anti‐Fas treatment as expected, but the LPS pre‐treated mice had significantly lower caspase‐8 activity upon anti‐Fas treatment (*P* < .001, Figure 1C), comparable to the control group, suggesting that the anti‐apoptotic effect was at the caspase‐8 level‐related events. Higher caspase‐8 activity was further confirmed by a significantly decreased level of Bid expression in livers of anti‐Fas‐treated mice when compared to the control or LPS pre‐treated group (*P* < .001 for both, Supporting Information, Figure S2).

### 3.2 The liver is infiltrated by inflammatory cells and pro‐inflammatory cytokines are induced upon LPS challenge

**Figure 2:**
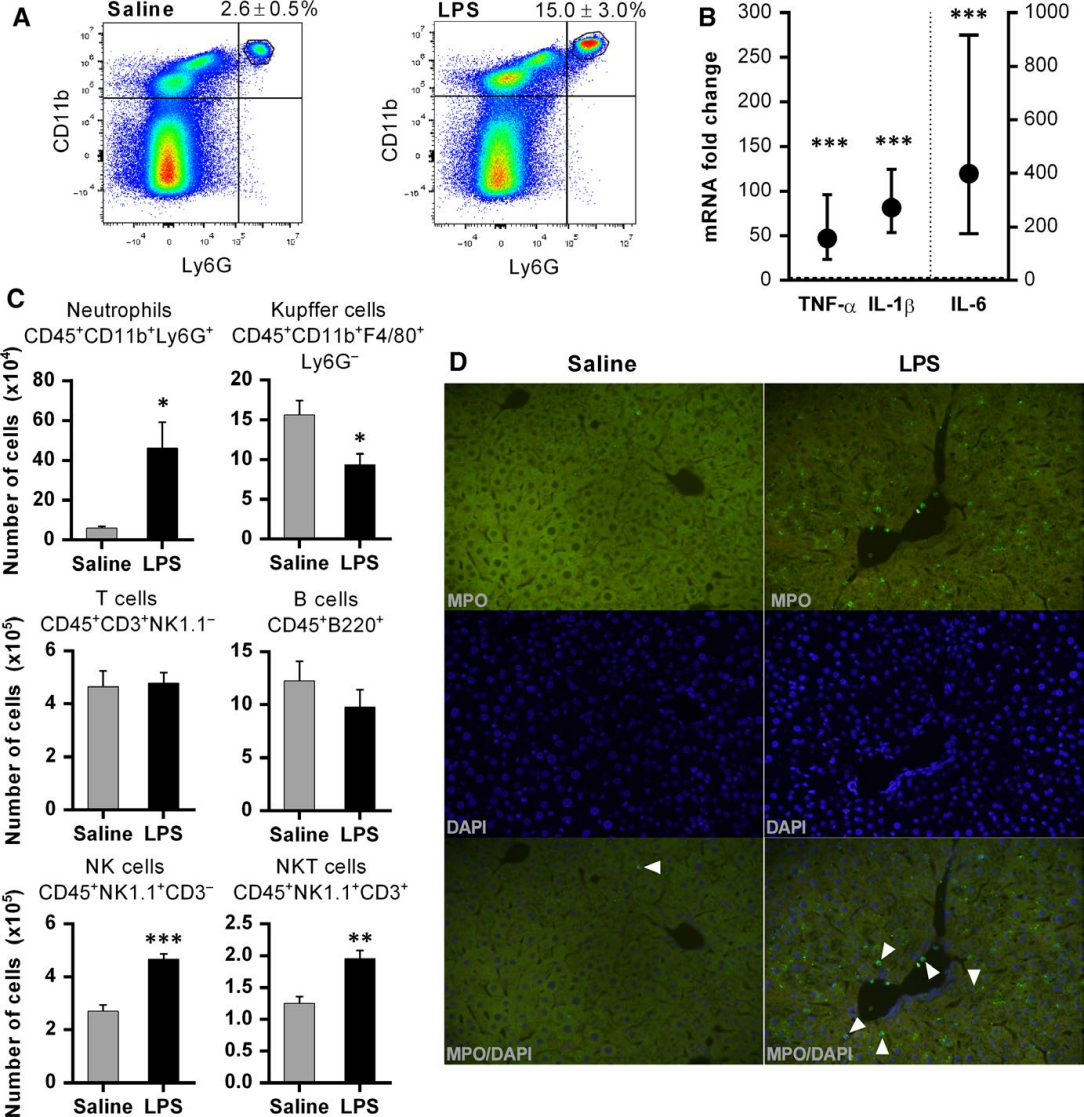
LPS treatment induces liver infiltration by inflammatory cells and up‐regulation of inflammatory mediators. Mice were treated with LPS (0.1 mg/kg) or saline. Two hours after the treatment, non‐parenchymal cells were isolated and analysed using flow cytometry, or liver specimens were sampled for gene expression assay. A, Representative flow cytometry data, showing the percentage of neutrophils, defined as CD45+ CD11b+ Ly6G+ cells in saline and LPS group. B, Analysis of gene expression showing up‐regulation of pro‐inflammatory cytokines in liver tissue upon LPS treatment. Symbols and bars represent fold change with 95% CI in comparison with control mice (horizontal dashed line). Secondary (right) *y*‐axis is associated with the data shown on the right side of the vertical dashed line. C, The absolute numbers of neutrophils, Kupffer cells, T cells, B cells, NK and NKT cells are shown and compared between saline and LPS groups. Columns and bars represent mean ± SEM; Student's *t* test. D, Immunohistochemical analysis confirms the increase of neutrophils in liver tissue following LPS treatment. White arrowheads indicate neutrophils that were determined by positive MPO staining (green) and morphological characteristics. DAPI (blue) was used to stain nuclei. n = 5 per group; *P < .05, **P < .01, ***P < .001 (saline vs LPS). CD, cluster of differentiation; DAPI, diamidino‐2‐phenylindole; IL, interleukin; LPS, lipopolysaccharide; MPO, myeloperoxidase; mRNA, messenger ribonucleic acid; NK, natural killer; TNF‐α, tumour necrosis factor‐alpha

To confirm that LPS induced an inflammatory response, we examined the changes in non‐parenchymal liver cells and gene expression of pro‐inflammatory cytokines in the liver after LPS treatment. Although the increase of overall cellularity per gram of liver tissue 2 hours after LPS did not reach significance (LPS = 3.5 ± 0.6 × 10^6^ vs saline = 3.1 ± 0.5 × 10^6^; *P* = .29), we noticed a significant increase in the number of neutrophils, NK and NKT cells (Figure 2A,C), with neutrophils showing the highest increase (7.6‐fold). This increase was further confirmed by immunohistochemistry (Figure 2D). There was no change in T‐ or B‐cell count, while Kupffer cell number exhibited a slight decrease (Figure 2C). As expected, LPS treatment also significantly induced gene expression of pro‐inflammatory cytokines in liver tissue, namely TNF‐α (*P* < .001), IL‐1β (*P* < .001) and IL‐6 (*P* < .001), as shown by Figure 2B.

### 3.3 LPS modulates the expression of pro‐inflammatory and apoptosis‐regulatory genes in liver

**Figure 3:**
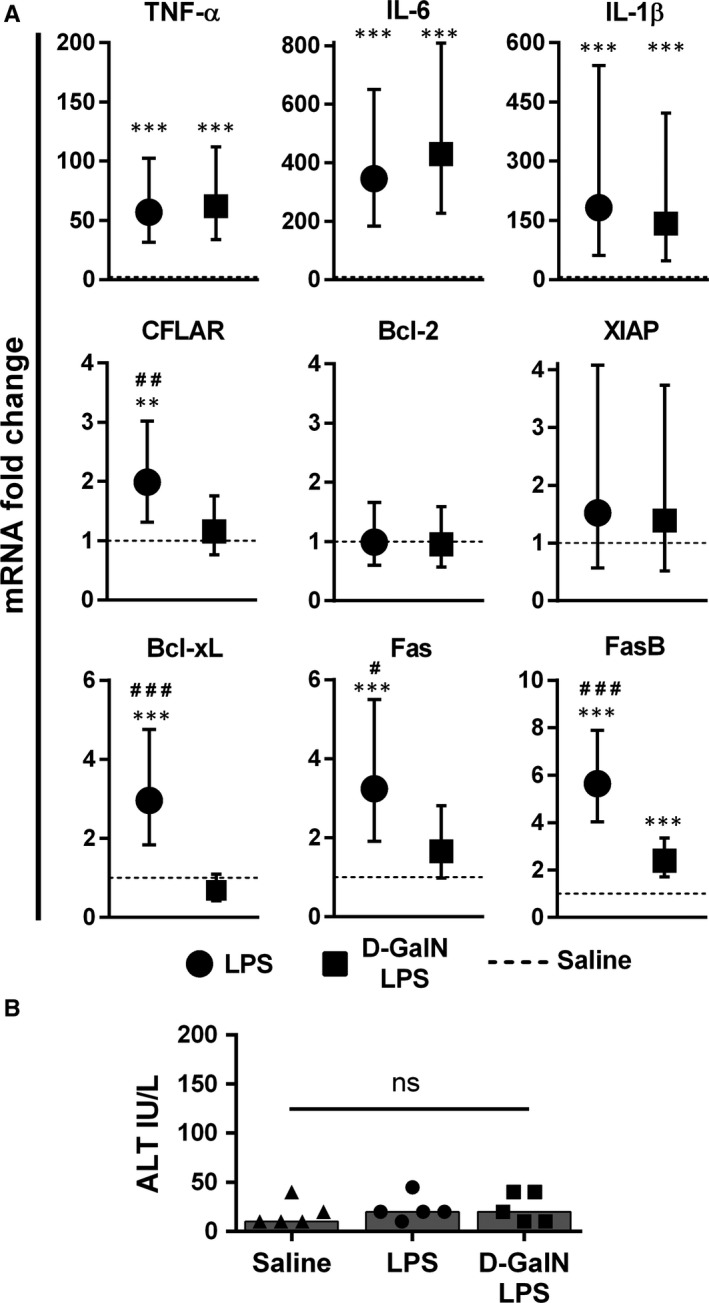
Gene expression analysis following LPS treatment. Mice were treated with LPS (0.1 mg/kg) with or without D‐GalN (700 mg/kg) pre‐treatment, while the control group received saline. A, The mRNA was isolated from liver specimens (collected 90 minutes after LPS or saline treatment) and analysed using PCR. Symbols and bars represent fold change with 95% CI in comparison with control mice (dashed line). All analyses were done at the ΔCt level using ANOVA with Bonferroni post hoc test. B, ALT levels were measured and compared between groups (sera collected 90 minutes after LPS or saline treatment). Symbols represent individual values, and columns represent medians. n = 5 per group; **P < .01, ***P < .001 (saline vs LPS, and saline vs D‐GalN/LPS); ^#^ *P* < .05, ^##^ *P* < .01, ^###^ *P* < .001 (LPS vs D‐GalN/LPS); ns—not significant. ALT, alanine aminotransferase; CI, confidence interval; Ct, cycle threshold; D‐GalN, d‐galactosamine; IL, interleukin; LPS, lipopolysaccharide; mRNA, messenger ribonucleic acid; PCR, polymerase chain reaction; TNF‐α, tumour necrosis factor‐alpha

In order to elucidate the possible mechanism beneath the anti‐apoptotic effect, we further analysed the LPS‐induced changes in the expression of pro‐inflammatory and apoptosis‐related factors in the liver. Beside the increased expression of pro‐inflammatory cytokines (TNF‐α, IL‐6 and IL‐1β), LPS enhanced the expression of CFLAR and Bcl‐xL, both known as apoptosis inhibitors (Figure 3A). There were no changes in Bcl‐2 and XIAP (Figure 3A). We also observed no changes in the gene expression of proteins involved in the cell cycle, such as cyclin D1 or survivin, upon LPS stimulus (Figure S3). However, an increase in both Fas and FasB expressions (soluble isoform) was observed, with former possibly favouring enhanced apoptosis and the latter possibly hampering apoptosis (Figure 3A).

To distinguish whether the observed changes occurred in hepatocytes or non‐parenchymal cells, the third group of animals received D‐GalN, a hepatocyte‐specific inhibitor of transcription, before LPS.^20^ As expected, TNF‐α, IL‐6 and IL‐1β were mainly induced in non‐parenchymal cells, and their expression was not suppressed by D‐GalN pre‐treatment (Figure 3A). On the other side, D‐GalN significantly blocked an increase in the expression of CFLAR, Bcl‐xL, Fas and FasB, suggesting that they are induced by LPS mainly in the hepatocytes (Figure 3A).

D‐GalN alone did not alter the gene expression in comparison with saline‐treated animals (data not shown). The 90‐minute time‐point was selected because the toxic effect of D‐GalN/LPS treatment would occur at a later time‐point. All serum ALT levels were within the normal range at the time of sampling, confirming that no toxic effect of D‐GalN/LPS treatment occurred (Figure 3B).

### 3.4 An increase in sFas expression in serum is not the mechanism of LPS anti‐apoptotic effect

The protein levels of sFas were undetectable in sera of saline or LPS‐treated mice (data not shown), ruling out the possibility that an increase of sFas due to shedding or alternative splicing might be the mechanism of protection.

### 3.5 Ruxolitinib pre‐treatment abrogates the protective effect of LPS‐induced acute inflammation on Fas‐mediated apoptosis

#### 3.5.1 LPS treatment induces phosphorylation of Stat3 in hepatocytes

**Figure 4:**
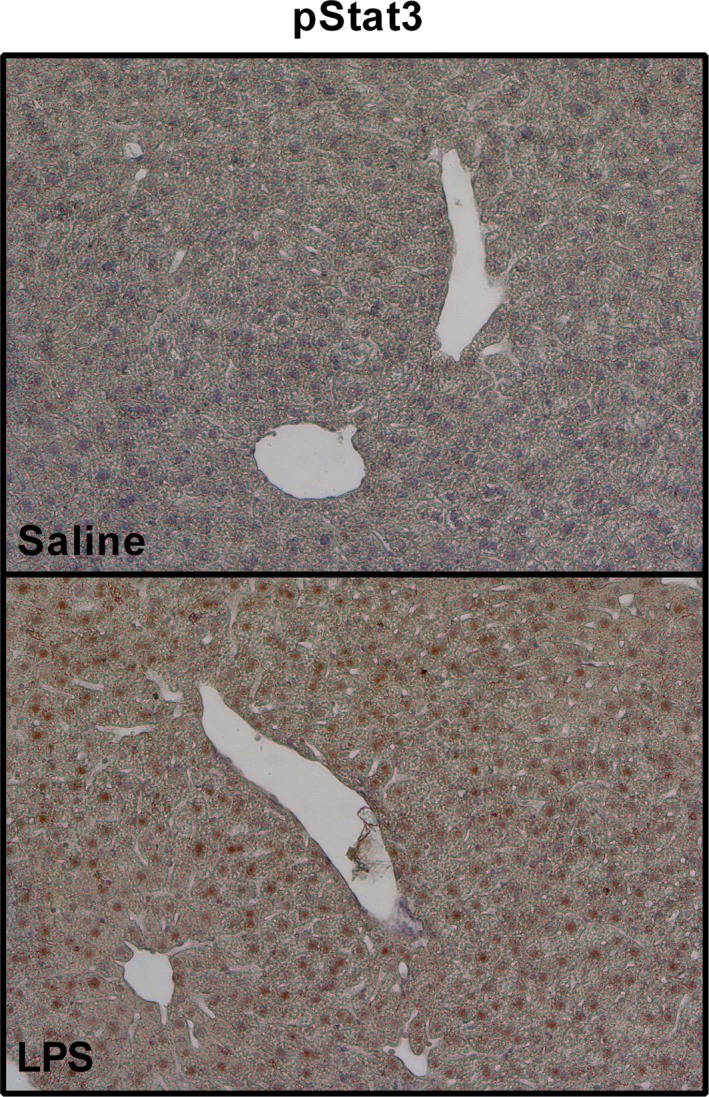
LPS induces Stat3 phosphorylation in hepatocytes. Immunohistochemical analysis showing the effect of LPS on Stat3 phosphorylation in hepatocytes. Mice were treated with LPS or saline, and liver samples were collected. LPS significantly induced pStat3 expression, as shown by brown staining of hepatocytes nuclei (anti‐phospho‐Stat3 with HRP‐conjugated secondary antibody). (p)Stat3, (phosphorylated) signal transducer and activator of transcription 3; HRP, horseradish peroxidase; LPS, lipopolysaccharide

Previous studies suggested Stat3 as one of the crucial mediators in the inflammation‐apoptosis interplay.^9^, ^10^ Therefore, we examined whether LPS treatment increases pStat3 in mice hepatocytes. Following LPS treatment, there was a remarkable increase in pStat3‐positive hepatocytes' nuclei (Figure 4).

#### 3.5.2 Ruxolitinib inhibits phosphorylation of Stat3 in hepatocytes

**Figure 5:**
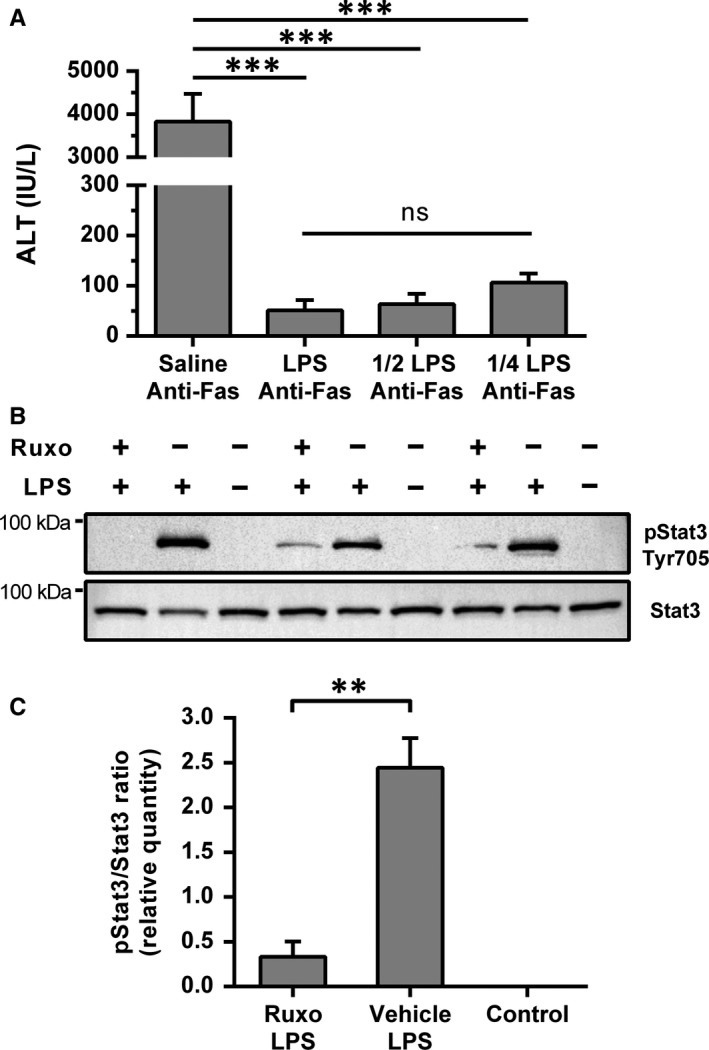
Ruxolitinib pre‐treatment inhibited LPS‐induced Stat3 phosphorylation in the liver. A, Dose effect of LPS treatment on anti‐Fas antibody induced apoptosis. Mice were treated with saline or LPS in three different doses: 0.1 mg/kg, 0.05 mg/kg and 0.025 mg/kg, followed by anti‐Fas antibody (JO2, 0.25 mg/kg) after 2 h. Sera were collected after 6 h, and ALT levels were analysed (n = 3‐4 per group). LPS = 0.1 mg/kg, 1/2 LPS = 0.05 mg/kg and 1/4 LPS = 0.025 mg/kg. Columns and bars represent mean ± SEM. ANOVA was used for analysis. B, Western blot analysis of pStat3 and Stat3 expressions in liver tissue. Mice were treated with ruxolitinib (180 mg/kg) or vehicle (DMSO + PEG300 + dH2O) followed by LPS (0.025 mg/kg) treatment 2 h after, and liver samples were collected 90 min after LPS treatment. The control group was treated with vehicle and saline. C, Analysis of pStat3 and Stat3 signal intensities, normalized to total protein signal intensity. Image Lab Software (Bio‐Rad) was utilized for signal intensity quantification and normalization. Columns and bars represent mean ± SEM of the pStat3/Stat3 signal intensity ratio, and Student's *t* test was used for the analysis (n = 3 per group). The stain‐free gels and the accompanying blotting membranes are shown in the Supporting information (Figure S4). **P < .01, ***P < .001, ns—not significant. (p)Stat3, (phosphorylated) signal transducer and activator of transcription 3; ALT, alanine aminotransferase; dH2O, distilled water; DMSO, dimethyl sulfoxide; LPS, lipopolysaccharide; PEG300, polyethylene glycol 300; Ruxo, ruxolitinib

To examine whether the Stat3 signalling pathway confers the anti‐apoptotic effect, we introduced ruxolitinib, a selective JAK1/2 inhibitor, to prevent Stat3 activation by LPS. Although we tried several different regiments of ruxolitinib application, we could not inhibit LPS‐induced Stat3 phosphorylation, so we hypothesized that inhibition could be achieved if we reduce the LPS stimulus. However, we first needed to ascertain that a lower dose of LPS still protects hepatocytes from Fas‐mediated apoptosis. Therefore, LPS was applied in 2 additional doses (0.05 and 0.025 mg/kg), and we found that protection is still present, even with a dose of 0.025 mg/kg (Figure 5A). Therefore, in subsequent experiments, the LPS dose of 0.025 mg/kg was used, and in those settings, ruxolitinib successfully prevented the phosphorylation of Stat3 in comparison with vehicle/LPS‐treated animals (Figure 5B). Immunoblots also revealed lower expression of Stat3 and significantly higher pStat3/Stat3 ratio in LPS‐treated mice in comparison with control mice or ruxolitinib/LPS‐treated mice (Figure 5C).

#### 3.5.3 LPS‐induced anti‐apoptotic effect is Stat3 dependent

**Figure 6:**
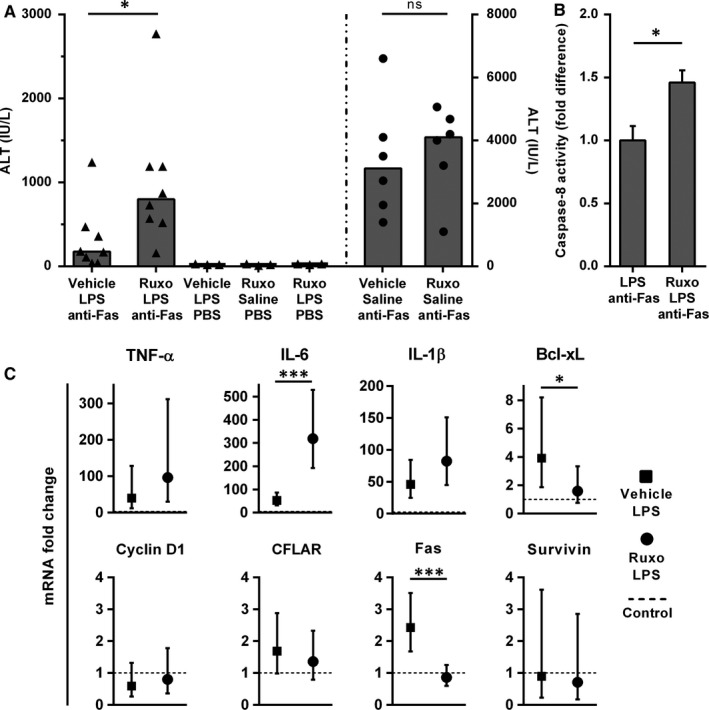
Ruxolitinib pre‐treatment reverses the protective effect of LPS‐induced acute inflammation on Fas‐mediated liver damage. Mice were treated with ruxolitinib (180 mg/kg) or vehicle (DMSO + PEG300 + dH2O), followed by LPS (0.025 mg/kg, or saline as a vehicle) and finally anti‐Fas antibody treatment (0.25 mg/kg, or PBS), after 2 and 4 hours, respectively. A, ALT levels in sera were determined 6 hours after anti‐Fas. Symbols represent individual values, and columns represent medians; Mann‐Whitney test. Secondary (right) *y*‐axis is associated with the data shown on the right side of the vertical dashed line. B, Caspase‐8 activity was determined in liver tissue by commercially available colorimetric assay. Columns and bars represent mean ± SEM; Student's *t* test. C, Gene expression analysis of pro‐inflammatory and apoptosis‐related factors. Animals received ruxolitinib (180 mg/kg) or vehicle, and LPS (0.025 mg/kg) was applied after 2 hours. After an additional 90 minutes, liver samples were collected, mRNA isolated and analysed using PCR. The control group was treated with vehicles. Symbols and bars represent fold change with 95% CI in comparison with control mice (dashed line). All analyses were done at the ΔCt level using ANOVA. *P < .05, ***P < .001, ns—not significant. ALT, alanine aminotransferase; CI, confidence interval; Ct, cycle threshold; dH2O, distilled water; DMSO, dimethyl sulfoxide; IL, interleukin; LPS, lipopolysaccharide; mRNA, messenger ribonucleic acid; PBS, phosphate buffered saline; PCR, polymerase chain reaction; PEG300, polyethylene glycol 300; Ruxo, ruxolitinib; TNF‐α, tumour necrosis factor‐alpha

To test whether inhibition of Stat3 phosphorylation abrogates LPS‐induced protection, we treated animals with ruxolitinib or vehicle, followed by LPS and anti‐Fas antibody. Ruxolitinib pre‐treatment significantly increased liver damage in these settings (*P* = .026; Figure 6A). Ruxolitinib/LPS/anti‐Fas group also exhibited significantly higher caspase‐8 activity, when compared to vehicle/LPS/anti‐Fas, suggesting that the caspase‐8 activation occurred in ruxolitinib pre‐treated animals (Figure 6B). Ruxolitinib or vehicle itself, or in combination with LPS, did not exhibit any detrimental effect on hepatocytes, as all ALT levels were within a normal range. Furthermore, hepatocyte apoptosis upon anti‐Fas treatment was not aggravated by ruxolitinib (*P* = .57, Figure 6A‐right axis).

### 3.6 Ruxolitinib pre‐treatment further increases LPS‐induced expression of pro‐inflammatory cytokines and modulates the expression of Bcl‐xL

As previous studies stated that JAK2 inhibitors could impair the production of specific pro‐inflammatory cytokines in LPS‐induced inflammation, such as IL‐6 and IL‐1β, we tested and compared the gene expression of cytokines and apoptosis‐related factors that were formerly altered by LPS treatment, between ruxolitinib/LPS and LPS‐only treated mice.^27^ Strikingly, gene expression of IL‐6 was significantly higher in ruxolitinib pre‐treated group when compared to LPS group (*P* < .001), while IL‐1β and TNF‐α were also increased but without reaching statistical significance (*P* = .06 and *P* = .14, respectively, Figure 6C), showing that these cytokines were not suppressed, but rather induced by ruxolitinib pre‐treatment upon LPS challenge. Furthermore, ruxolitinib pre‐treatment reduced the LPS‐induced expression of apoptosis inhibitor Bcl‐xL when compared to LPS‐treated animals (*P* = .02, Figure 6C), while CFLAR expression was not significantly altered. The inhibitory effect of ruxolitinib on LPS‐induced protection from apoptosis was not mediated through an increase in Fas expression, as the ruxolitinib pre‐treated group exhibited lower Fas expression when compared to LPS‐treated animals (*P* < .001, Figure 6C).

## 4 DISCUSSION

Although several recent reports have suggested that the induction of the TNF‐α, a soluble mediator, or cellular mediators in inflammation could promote hepatocyte death following Fas activation,^1^, ^2^, ^3^, ^28^ in the present study, we show that the net effect of LPS inflammation is anti‐apoptotic in terms of Fas‐mediated apoptosis. The anti‐apoptotic effect of LPS can be inhibited through pharmaceutical inhibition of the JAK/Stat3 signalling pathway by ruxolitinib. Ruxolitinib itself had no detrimental effect nor did it exaggerate the apoptosis when applied in combination with anti‐Fas.

The applied dose of LPS has effectively increased several pro‐inflammatory mediators implicated to promote apoptosis either through crosstalk with Fas signalling pathway (TNF‐α) or through an expression of FasL (by neutrophils, NK and/or NKT cells).^3^, ^28^, ^29^ These mediators are also capable of inducing liver injury through pathways other than Fas/FasL.^29^ However, through our experiments, we show that the overall effect of LPS‐induced inflammation is the desensitization of hepatocytes to Fas‐mediated apoptosis. Considering previous reports on anti‐apoptotic effects of other cytokines released in inflammation, such as IL‐6 or IL‐22,^9^, ^10^ our results indicate that a net result of complex and often confronting signals activated in hepatocytes during the inflammation is their shift towards the anti‐apoptotic state.

To further elucidate the effect of inflammation on hepatocyte apoptosis, we focused our attention on cellular responses activated in hepatocytes rather than on one specific cytokine. We first showed that two crucial anti‐apoptotic molecules Bcl‐xL and CFLAR are induced specifically in hepatocytes during the inflammation. As Haga et al^30^ reported that both molecules are up‐regulated by Stat3 in hepatocytes, we further studied its activation and found increased phosphorylation upon inflammatory stimulus. To show the causal relationship between the Stat3 phosphorylation and resistance to apoptosis, we pre‐treated mice with ruxolitinib and confirmed that it indeed significantly abrogates the protective effect of LPS‐induced inflammation on Fas‐mediated apoptosis. Furthermore, ruxolitinib pre‐treatment blocked the up‐regulation of Bcl‐xL, while there was no difference in CFLAR expression between ruxolitinib/LPS and LPS‐only treated animals. This hallmarks the Bcl‐xL as a molecule that might play a pivotal role in inflammation‐apoptosis interplay. It was previously reported that Bcl‐xL could block caspase‐3 and caspase‐8 activation in type II cells^31^ or even mediate the neutralization of active caspase‐8.^32^ When its inhibitory effect on mitochondrial events is also considered,^33^ it is comprehensible that Bcl‐xL up‐regulation might have hampered the apoptotic processes. However, the possible contribution of Bcl‐xL or CFLAR warrants further investigation.

Previous studies suggested that JAKs inhibition could impair the LPS‐induced activation of macrophages and the production of IL‐6 and IL‐1β.^27^, ^34^ However, those studies used macrophage‐like cell lines or peritoneal macrophages, and both applied AG490 to inhibit JAK2. In contrast, we found that ruxolitinib pre‐treatment with subsequent LPS administration further up‐regulated IL‐6 and IL‐1β expressions, excluding the possibility that apoptosis sensitization effect might occur due to anti‐inflammatory properties of ruxolitinib. These discrepancies might emerge due to differences between Kupffer cells and macrophage cultures or peritoneal macrophages or due to lower specificity of AG490 inhibitor that can also inhibit epidermal growth factor receptor signalling, essential for LPS‐induced signalling, and IL‐6 and TNF‐α production.^35^, ^36^, ^37^, ^38^

So far, ruxolitinib has been typically used in chronic models with multiple applications, and we have explored its effect in an acute experimental model. We have confirmed its effect by immunoblotting of pStat3. However, this study has some limitations, and the results should be cautiously interpreted. Ruxolitinib pre‐treatment has not completely abolished the anti‐apoptotic effect of LPS‐induced inflammation, and there are several possible explanations for the residual protection. There could be additional JAK/Stat3‐independent mechanisms that are induced by LPS and support the anti‐apoptotic response, such as NF‐κB activation.^39^ However, recent research has proposed a completely different role for NF‐κB activation in hepatocytes, suggesting that it is one of the crucial mechanisms in sensitization to apoptosis rather than protecting from it,^3^ and further research is warranted to resolve this complex issue. Nevertheless, ruxolitinib might also not entirely suppress JAK/Stat3 in hepatocytes, or Stat3 might be phosphorylated at later time‐points, as it is described that LPS shows the ability to activate Stat3 for up to 8 hours after treatment.^40^

Levels of sFas in plasma, either secreted or shedded from the membrane, play an important anti‐apoptotic mechanism in human pathophysiology.^41^ However, this role in mice seems to be negligible.^42^ Nevertheless, Hughes and Crispe^43^ reported a naturally occurring sFas isoform FasB that is created through alternative splicing in mice. Furthermore, a shedding process of the membrane‐bound Fas receptor might also arise during inflammation.^44^ However, the up‐regulation of FasB that we noticed at the gene level was not followed by an increase in serum concentration, ruling out the probability that the anti‐apoptotic effect emerged due to sFas production or shedding.

TLR4 signalling and Stat3 are suggested to be involved in hepatocarcinogenesis and tumour progression, and TLR4 signalling was shown to promote HCC proliferation in a Stat3‐dependent manner.^17^, ^18^, ^45^ Therefore, the anti‐apoptotic effect of inflammation that we report here might be beneficial in conditions where apoptosis contributes to liver injury. Still, on the other hand, it might also be involved in HCC development and progression by contributing to apoptosis resistance of cancer cells. Ruxolitinib treatment might potentially reverse these effects, and indeed, some reports suggested that ruxolitinib may inhibit the development of liver tumours.^46^, ^47^ On the other hand, our data raise a concern that ruxolitinib therapy might aggravate liver injury in patients with liver‐damaging conditions. To confirm or repudiate these considerations, clinical studies on a larger scale should be conducted.

In summary, although recent reports have revealed the pro‐apoptotic effects of isolated pro‐inflammatory mediators, such as TNF‐α, the overall impact of inflammatory milieu on hepatocytes is Stat3‐dependent desensitization to apoptosis. As gut‐derived LPS is now considered to be involved in the development and progression of various liver diseases, further research and efforts are warranted to elucidate the delicate balance between apoptosis, inflammation and regenerative processes in the liver.

## CONFLICT OF INTEREST

The authors confirm that there are no conflicts of interest.

## AUTHOR CONTRIBUTIONS

AM, IC and TK designed the research study. AM, DF and TK performed the experiments and analysed the data. DG and NK performed, analysed and interpreted the flow cytometry data and critically revised the manuscript. AS and NL performed the gene expression assays, analysed and interpreted the gene expression data. HL and NL performed and analysed protein expression data. KV and PT performed, analysed and interpreted histology and immunohistochemistry data. AM, DF and TK wrote the manuscript. DP and IC contributed essential reagents and critically revised the manuscript. All authors have revised and edited the manuscript and approved it for submission.

## Data Availability

The data that support the findings of this study are available from the corresponding author upon reasonable request.
