# Supplementary material for: LPS‐induced inflammation desensitizes hepatocytes to Fas‐induced apoptosis through Stat3 activation—The effect can be reversed by ruxolitinib
Source: J Cell Mol Med. 2020 Feb 5;24(5):2981–92. doi: 10.1111/jcmm.14930 (PMC7077556; doi:10.1111/jcmm.14930)
Supplement: Supplementary file 1 [file JCMM-24-2981-s001.pdf]

SUPPORTING INFORMATION

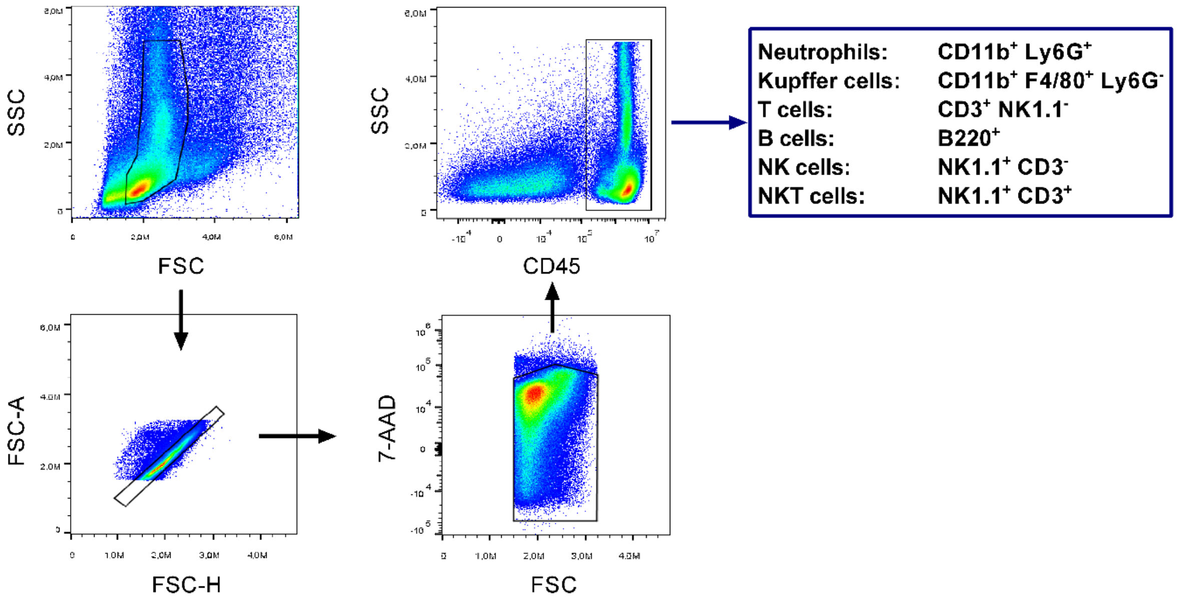

Figure S1 The gating strategy for flow cytometric analysis of non-parenchymal liver cells. Representative flow cytometry data showing the gating strategy. Single, live (7AADneg), hematopoietic (CD45+) cells were included in the analysis and populations of interest were defined as designated in the blue box.

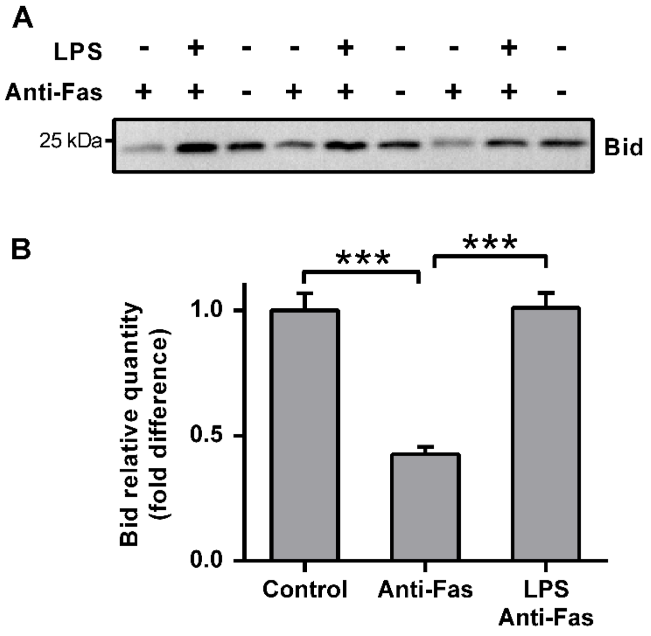

Figure S2 Protein levels of Bid in mice livers following the anti-Fas treatment.

Mice were treated with LPS (0.1 mg/kg) or saline and after 2 hours the activating anti-Fas antibody (0.25 mg/kg) or vehicle (PBS) was injected. The control group received vehicles (saline and sterile PBS). Liver specimens were collected 6 hours after anti-Fas treatment. (A) Protein levels of Bid were determined in the cytoplasm of liver cells by western blot. (B) Analysis of Bid signal intensity, normalized to the total protein signal intensity, across groups. Image Lab Software (Bio-Rad) was utilized for signal intensity quantification and normalization. Columns and bars represent mean ± SEM of the Bid relative quantity, and ANOVA was used for the analysis (n=3 per group). The Stain-Free gels and the accompanying blotting membranes are shown below (Figure S4).

***p<0.001. Abbreviations: LPS lipopolysaccharide, PBS phosphate buffered saline.

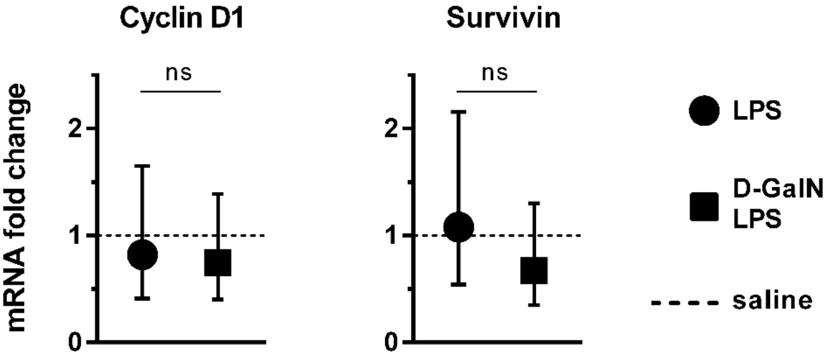

Figure S3 Gene expression of cyclin D1 and survivin following the LPS treatment. Mice were treated with LPS (0.1 mg/kg) with or without D-GalN (700 mg/kg) pretreatment, and the control group received saline. Following mRNA isolation from liver tissue specimens, PCR analysis was performed and gene expression of cyclin D1 and survivin was determined. Symbols and bars represent fold change with 95% CI in comparison with control mice (dashed line). Analyses were done at the ΔCt level using ANOVA with Bonferroni correction.

Abbreviations: mRNA messenger ribonucleic acid, LPS lipopolysaccharide, D-GalN d-galactosamine, PCR polymerase chain reaction, CI confidence interval, Ct cycle threshold, ns - not significant.

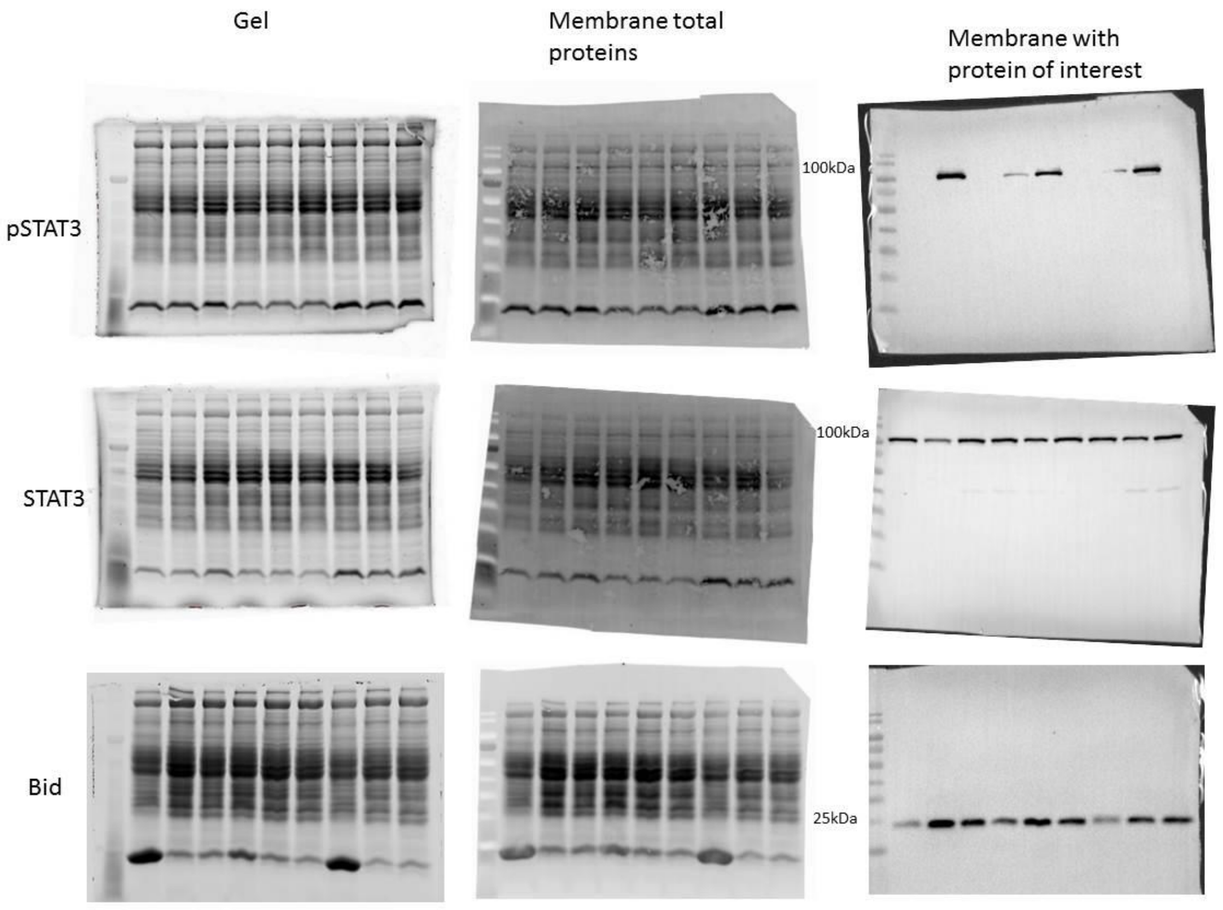

Figure S4 Western blot gels and membranes.

The gels and membranes used for detection and quantification of total proteins and the proteins of interest (pStat3, Stat3 and Bid) are shown. Stain-Free technology was utilized as loading control.

TaqMan assays IDs:

TNF-α (Mm00443258_m1)

IL-6 (Mm00446190_m1)

IL-1β (Mm00434228_m1)

Fas (Mm01204968_m1)

Bcl-2 (Mm00477631_m1)

Bcl-xL (Mm00437783_m1)

CFLAR (Mm01255578_m1)

XIAP (Mm00776505_m1)

Cyclin D1 (Mm00432359_m1)

Survivin (Mm00599749_m1)

GAPDH (Mm99999915_g1)
